# Plant-Based Bioactive Molecules in Improving Health and Preventing Lifestyle Diseases

**DOI:** 10.3390/ijms22062991

**Published:** 2021-03-15

**Authors:** Rosaria Acquaviva, Giuseppe Antonio Malfa, Claudia Di Giacomo

**Affiliations:** Department of Drug and Health Sciences, Section of Biochemistry, University of Catania, Viale A. Doria 6, 95125 Catania, Italy; g.malfa@unict.it (G.A.M.); cdigiaco@unict.it (C.D.G.)

The Special Issue, “Plant-Based Bioactive Molecules in Improving Health and Preventing Life-style Diseases”, includes original research papers and reviews, which aim to increase knowledge of the molecular mechanisms underlying multiple biological effects of natural compounds from plants, responsible for maintaining human health and improving many diseases caused by people’s daily lifestyles. It is expected that this Special Issue will be useful for researchers and scholars involved in the studies regarding plant secondary metabolites and their biological activities. 

It is widely demonstrated that the change in people’s behavior or lifestyles, oriented towards a healthy and diversified diet associated with moderate physical activity and by breaking down unhealthy habits, significantly reduces the risk of numerous diseases such as cardiovascular disease, diabetes, cancer, etc. Obviously, all diseases, including the so-called lifestyle diseases, have multiple causes and it would be simplistic to charge the entire prevention strategy to the correct lifestyle alone; however, there is no doubt that a diet based on a diversified source of vegetables reduces the incidence of numerous human disorders and diseases. From immemorial times, many vegetables are known to have been used to exert healing properties against human ailments due to their content of secondary active metabolites. In recent years, biological activities, nutritional values, and potential health and therapeutic benefits of several medicinal and edible plants have been intensively explored and investigated and their role in the prevention and mitigation of lifestyle-related disorders has been widely confirmed. In particular, many foods of plant origin and herbal remedies, typical of the tradition of many cultures around the world, possess innumerable beneficial effects. In this Special Issue, the diverse health functions and the various mechanisms of actions of the major bioactive components of tea (*Camelia sinensis*), one of the most popular beverages all over the world, have been widely demonstrated by several in vitro, in vivo, and human studies [1]. Seaweed polysaccharides, often recommended as food and food additives, have also been reviewed for their multiple health functions and effectiveness as antioxidants, immune-modulatory, anti-inflammatory, anti-coagulant, and anticancer agents [2]. Besides their well-known antioxidant and pro-oxidant activities, natural compounds have been also reported to be involved in anti-mutagenic and/or anti-carcinogenic pathways and to possess several other biological activities. In particular, Costantini et al. showed that the hydroxytyrosol contained in olive oil (*Olea europea*) induces the apoptotic death of melanoma cells by increasing ROS levels, p53 and γH2AX expression and by the decrease in AKT expression [3]. A Thai herbal preparation known as Benja Amarit is capable of inducing apoptotic death in cholangiocarcinoma cells, through the regulation of the nuclear factor-KB (NF-KB) signaling pathway [4]. Both hydroxytyrosol and Benja Amarit preparation result in safe and promising therapeutic tools for the treatment of these highly invasive tumors.

The study of Chang et al. confirmed that some phytochemical compounds, such as berberine, an isoquinoline alkaloid, and its derivatives, can also be considered as therapeutic agents for non-small cell lung cancer for their ability to affect cell cycle regulation, thereby causing growth inhibition and tumorigenesis suppression [5].

The cytotoxic activity of plant secondary metabolites in cancer is also related to their anti-inflammatory effects. *Artocarpus lakoocha* Roxb, known for its high content of stilbenoids, especially oxyresveratrol, decreases production and secretion of cytokines and chemokine, including IL-6, TNF-α, and inhibits the production of nitric oxide (NO) in RAW cells. The anti-inflammatory effects are mainly due to the negative modulation of PI3K/ Akt and NF-kB signal transduction pathways [6].

The anti-inflammatory effect of the stilbenoid oxyresveratrol was also observed in the human microglial cell line HMC3. The effect is mediated by suppressing the PI3K/AKT/p70S6K and ERK1/2 MAPK signaling pathways [7].

Neurological-related disorders are seen as increasingly important aspects of welfare. It is reported that a diet containing polyphenols, associated to regular exercise, represents an effective non-pharmacological approach that prevents the progression of neurodegenerative diseases. Broderick et al. confirmed that resveratrol decreased neuroinflammation and accumulation of Aβ oligomers, increased levels of neurotrophins and decreased markers of apoptosis, autophagy, endolysosomal degradation and ubiquitination in mouse brains in an in vivo model of Alzheimer’s disease [8].

Many clinical studies have reported that neurodegenerative disorders such as Alzheimer’s disease are characterized by microglial hyperactivation and neuroinflammation; Lee et al. demonstrated that *Aquilariae lignum*, called agarwood, attenuated the LPS-induced overproductions of NO, cyclooxygenase-2 (COX-2), prostaglandin E2 (PGE2), and IL-1α in BV2 microglial cells. These anti-inflammatory effects were supported by the modulation of the NF-KB pathway [9].

Neuroprotective effects of natural compounds were confirmed by the study of Landucci and collaborators that suggested that both *Panax ginseng* and *Ginkgo biloba* extracts may mediate their effects by activating phosphorylation of ERK1/2 and Akt signaling pathways, so protecting against excitotoxicity-induced damage [10].

Natural sesquiterpenes, such as (E)-caryophyllene (BCP), present in the essential oil of different plant species, possess several important pharmacological activities, ranging from pain treatment to neurological and metabolic disorders. These effects are mainly due to their ability to interact with the cannabinoid receptor 2 (CB2) and the complete lack of interaction with the brain CB1 [11].

BCP has been reported to be active against several disorders, with particular reference to chronic pain, inflammation and cancer. Moreover, the specificity of BCP for the CB2 receptor, mainly expressed in peripheral tissues, and its inability to bind CB1 implies that its action is devoid of the known psychoactive effects associated with the activation of CB1. So, BCP may be an interesting alternative to the use of Cannabis.

It is known that the inflammation is also related to the metabolic syndrome, including diabetes, because a chronic inflammatory state contributes to development of insulin resistance and progressive loss of cell function and mass. Novelli et al. reported that the extract of *Hypericum perforatum* (St. John’s wort, SJW) and hyperforin exert protective effects hindering inflammatory cytokine signaling and activating the cyclic adenosine monophosphate (cAMP)/protein kinase cAMP-dependent (PKA)/adenosine monophosphate-activated protein kinase (AMPK) pathway [12].

Since diabetes, hypertension and cardiovascular disease are additional risk factors for thrombosis that is also associated to oxidative stress, the dietary supplement with natural compounds such as flavonoids, isoflavonoids, and terpenoids might be useful to reduce the risk of thrombosis reducing ROS levels and pro-inflammatory cytokines; in addition, they exercise anticoagulant effects, probably via the endogenous coagulation system and thus inhibit platelet aggregation/function or prothrombotic effects [13].

Medicinal plants have historically proven their value as a source of molecules with therapeutic potential; nowadays they still represent not only an important pool for the identification of novel drugs such as taxol or vincristine but also to detect chemical compounds to radiosensitize cancer cells so to make them more responsive to conventional antineoplastic treatment such as radiotherapy.

It is known that use of curcumin, resveratrol, withaferin A (a steroidal lactone), celastrol (a pentacyclic triterpenoid) isolated from the root of the “thunder god vine” (*Tripterygium wilfordii*), ursolic acid, and zerumbone, isolated from rhizomes of *Zingiber zerumbet*, can counteract the mechanisms of tumor resistance to radiotherapy with different action mechanisms such as increasing ROS production, inhibition of HIF-1 protein levels and inducing a reduction of the intratumoral necrotic areas [14].

Plants, such as *Allium sativum*, *Citrus aurantifolia*, *Ipomoea aquatic*, *Malasezzia restricta*, *Malasezzia globosa*, *Malasezzia sympodialis*, *Malasezzia furfur*, and *Ficus sycomorus* can act as immunomodulators of the cutaneous response and possess both anti-inflammatory and pro-inflammatory properties. Plant extracts have been found to have inhibitory effects on the growth of bacteria, viruses, and fungi in in vitro/in vivo studies. Their activities are frequently related to the blockage of the synthesis or function of key components of particular organisms. Moreover, natural compounds may interfere with bacterial resistance mechanisms, disrupting viral attachment and entry into the host [15]. From these studies, it is evident that plant secondary metabolites demonstrate several reliable biological activities against many diseases and related complications. Certainly, preclinical and clinical research are needed to better understand their pharmacological action, safety and efficacy.

## Data Availability

Not applicable.
